# Modes of Effective Connectivity within Cortical Pathways Are Distinguished for Different Categories of Visual Context: An fMRI Study

**DOI:** 10.3389/fnbeh.2017.00064

**Published:** 2017-05-23

**Authors:** Qiong Wu, Jinglong Wu, Shigeko Takahashi, Qiang Huang, Hongzan Sun, Qiyong Guo, Yoshio Ohtani, Yoshimichi Ejima, Xu Zhang, Chunlin Li, Tianyi Yan

**Affiliations:** ^1^Key Laboratory of Biomimetic Robots and System, Ministry of Education, State Key Laboratory of Intelligent Control and Decision of Complex Systems, Beijing Institute of TechnologyBeijing, China; ^2^Cognitive Neuroscience Laboratory, Graduate School of Natural Science and Technology, Okayama UniversityOkayama, Japan; ^3^Kyoto City University of ArtsKyoto, Japan; ^4^Key Laboratory of Biomimetic Robots and Systems, Ministry of Education, Beijing Institute of TechnologyBeijing, China; ^5^Department of Radiology, Shengjing Hospital of China Medical UniversityShenyang, China; ^6^Faculty of Engineering and Design, Kyoto Institute of TechnologyKyoto, Japan; ^7^School of Biomedical Engineering, Capital Medical UniversityBeijing, China; ^8^School of Life Science, Beijing Institute of TechnologyBeijing, China; ^9^Key Laboratory of Convergence Medical Engineering System and Healthcare Technology, The Ministry of Industry and Information Technology, Beijing Institute of TechnologyBeijing, China

**Keywords:** contextual information, color context, shape context, 3D-depth context, fMRI, dynamic causal modeling

## Abstract

Context contributes to accurate and efficient information processing. To reveal the dynamics of the neural mechanisms that underlie the processing of visual contexts during the recognition of color, shape, and 3D structure of objects, we carried out functional magnetic resonance imaging (fMRI) of subjects while judging the contextual validity of the three visual contexts. Our results demonstrated that the modes of effective connectivity in the cortical pathways, as well as the patterns of activation in these pathways, were dynamical depending on the nature of the visual contexts. While the fusiform gyrus, superior parietal lobe, and inferior prefrontal gyrus were activated by the three visual contexts, the temporal and parahippocampal gyrus/Amygdala (PHG/Amg) cortices were activated only by the color context. We further carried out dynamic causal modeling (DCM) analysis and revealed the nature of the effective connectivity involved in the three contextual information processing. DCM showed that there were dynamic connections and collaborations among the brain regions belonging to the previously identified ventral and dorsal visual pathways.

## Introduction

Context contributes to accurate and efficient information processing, thoughts and actions. Along with predictive coding, which is a guiding principle of efficient information processing in the brain, contextual information allows for context-driven predictions or expectations. Thus, humans can recognize thousands of objects in a cluttered scene, despite variability in pose or changes in object occlusion. However, little is known about the dynamics of the neural mechanisms that support these abilities.

Previous studies (Rugg et al., 1999; Rüttiger et al., 1999; Scholl and Pylyshyn, 1999) have shown that visual contextual processing is carried out hierarchically in several brain domains. Within the spatial domains, one type of contextual information is the feature context, which is the “glue” that binds visual features to coherent objects and scenes. Neural sites responsible for feature context processing include the inferior frontal gyrus (IFG), inferior temporal gyrus (ITG), inferior parietal lobe (IPL), and postcentral gyrus (PG) (Kwon et al., 2016).

Another type of contextual information is the association context of object and color, which plays a role in object recognition based on prior knowledge (memory color). Recent functional magnetic resonance imaging (fMRI) studies demonstrate that the parahippocampal gyrus (PHG), IFG and ITG contribute to the processing of association context (Kourtzi and Kanwisher, 2000; Haxby et al., 2001; Boccia et al., 2016; Kwon et al., 2016). The feature and association contexts allow the visual system to sensitize the corresponding visual representations for easier recognition of the adjacent objects once attended. In this sense, the attentional system is closely related to the contextual information processing system. It has been shown that the visual attentional system is a network that consists of early visual regions, such as Brodmann's areas 17/18/19, fusiform gyrus (FG), superior/inferior parietal cortex (SPL/IPL) and frontal eye fields (FEF) (Coull and Nobre, 1998; Nobre et al., 1998). Activation of these neural regions has been proposed to be relied on an intrinsic interplay between exogenous and endogenous sources of information in then distinctly distributed neural networks in the lateral prefrontal cortex (PFC) (Miller and Cohen, 2001; Rowe et al., 2005). However, to better understand the dynamics of the mechanisms involved in these neural sites and networks, anatomically precise models are needed to define how the connections change with context.

**Figure 1:**
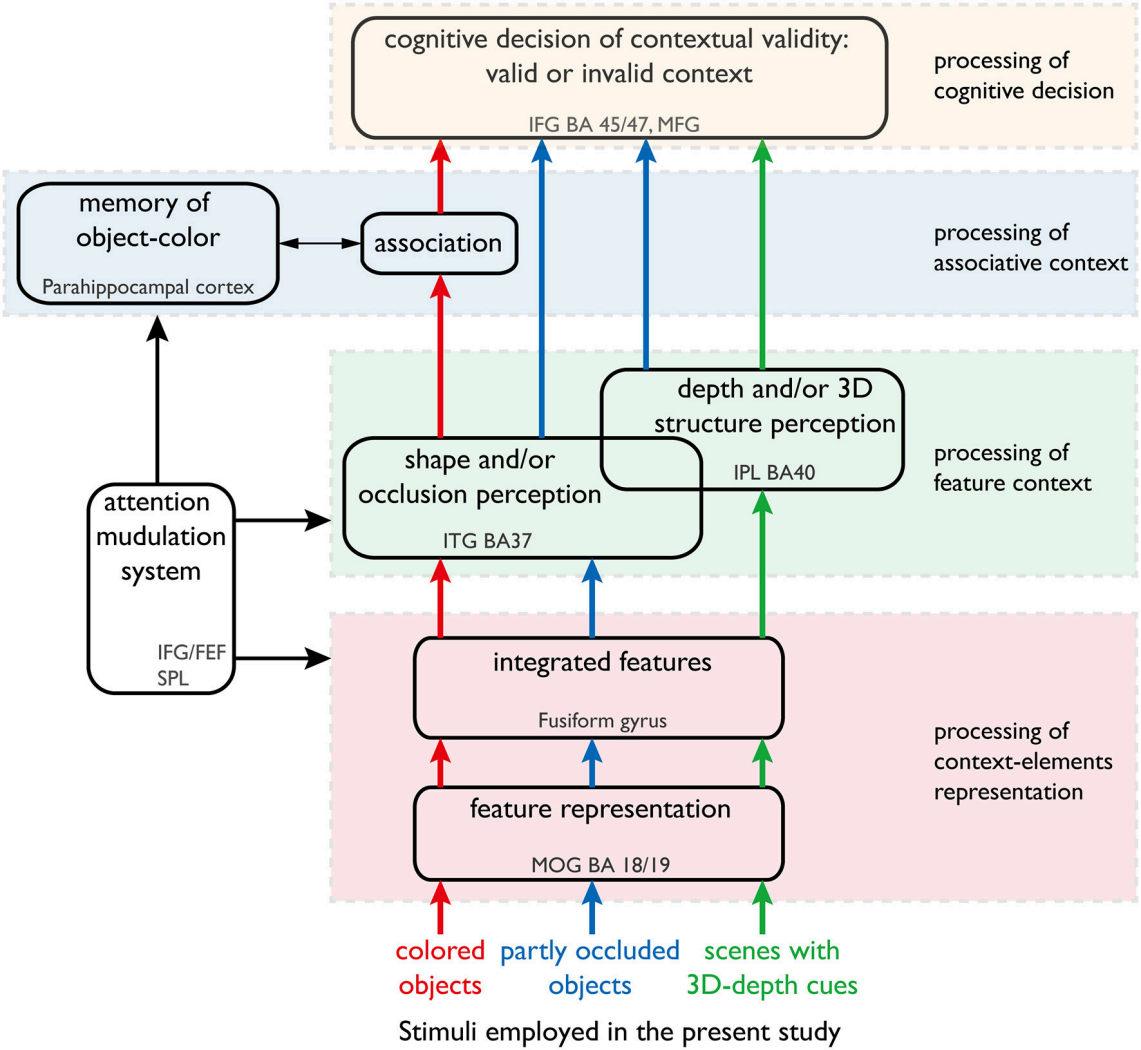
**Our hypothesis of the neural networks responsible for processing color, shape and 3D-depth contexts**. This hypothesis was used to design the fMRI experiment. The arrows denote the information streams. The colored (red, blue, and green) arrows denote the information streams for color, shape, and 3D-depth cues. The solid oblong shapes denote the type of information processing or function of the neural networks. The dotted rectangles denote the cortical sites that are thought to sub-serve that type of information processing or function.

In the present study, we focused on the dynamics of the neural mechanisms underlying accurate object recognition despite variability in the visual contexts (Figure 1). We analyzed the dynamics by imaging the neural activations of subjects while responding to the objects of different color, shape and 3D-depth. The findings provide new evidence for existence of subnetworks within the recognized visual context processing networks and further our understanding of neural dynamics.

## Methods

### Subjects

Twenty-one subjects were enrolled. The subjects were all right-handed, with a means ±SD age of 22 ± 2.6 years (14 females and 7 males). All subject had no history of neurological or psychiatric illness, and had normal or corrected-to-normal vision. All procedures were approved by the ethics committee of China Medical University (Human Studies Protocol number 2010PS11) in accordance with the declaration of Helsinki (2008). Informed written consent was obtained from each of the subjects before the scanning session.

### Stimuli

**Figure 2:**
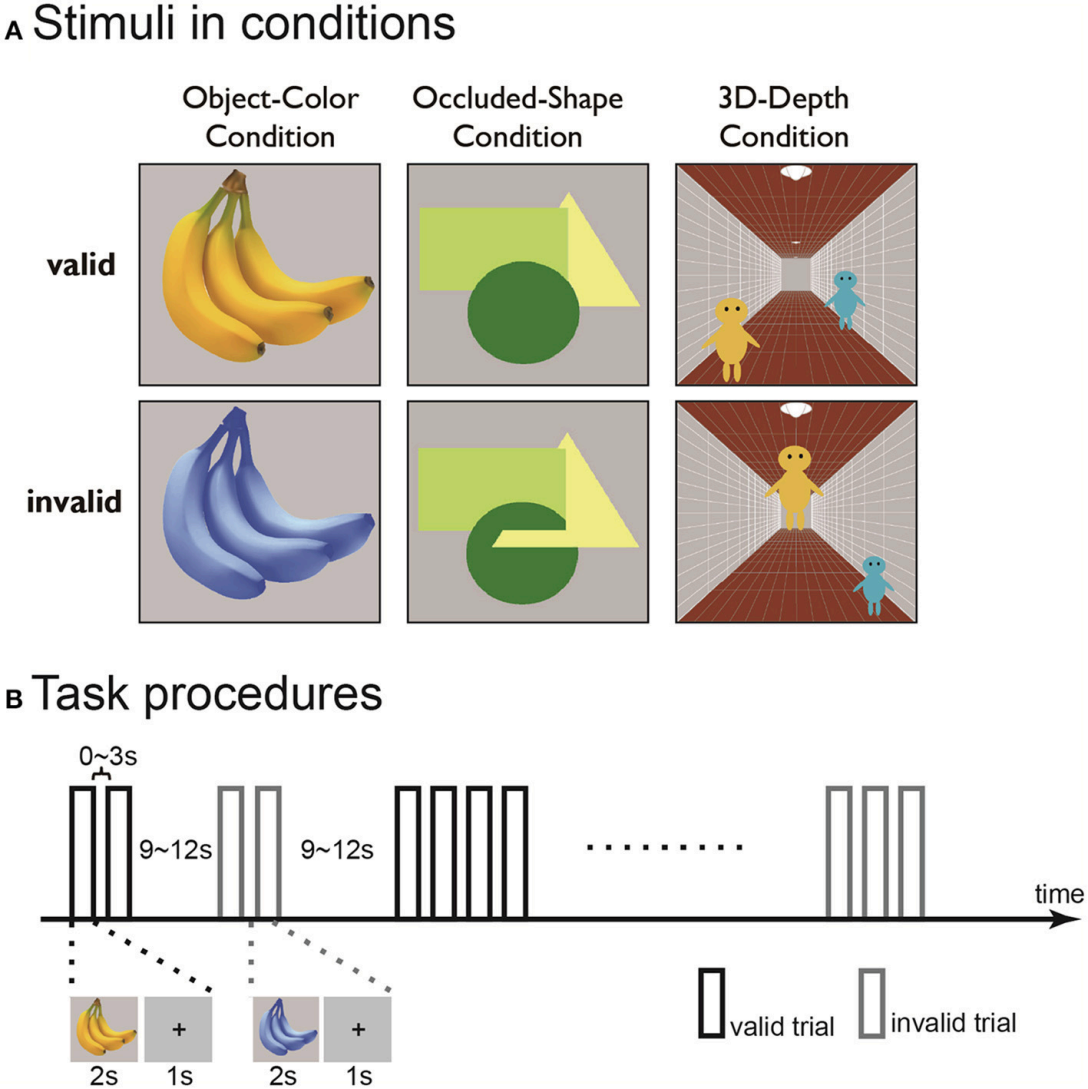
**Examples (A)** and time course **(B)** of the contextually valid (left) and invalid (right) visual stimuli for color (top), shape (middle), and 3D-depth (bottom) contexts used in the study.

We employed stimuli with valid and invalid contexts to identify the brain regions involved in the visual association processing of objects of different color, shape and 3D-depth (Figure 2A). In order to find the modulatory effect, we employed a pair of contrast conditions for each context: natural vs. unnatural color, interwoven vs. stacked shape and normal vs. abnormal 3D scene. For each set of objects, 50 valid or invalid stimuli were presented.

For color stimuli, photos of fruits and vegetables were used. The invalid images were prepared by changing the CMYK composition of the photos while maintaining a constant yellow color across the photos. The objects were presented at the center of a visual field on gray background at a visual field of 8° × 8°.

For shape stimuli, three geometric figures a circle (green), an equilateral triangle (yellow) and a square (light-green) were used to interweave each other. For the valid stimuli, the figures were stacked but not interwoven, while for the invalid stimuli, the figures were interwoven such that the original shapes of the figures were disrupted, giving rise to the impression that one part of an object is in front of another and/or that the objects have complex shapes embedded in each other like a jigsaw puzzle. The objects were presented as described above.

For 3D-depth conditions, images with three pictorial depth cues were used. The cues included linear perspective cue (a corridor), a size cue (two people, assumed to have identical body size) and a height-in-field cue (the positions of the two people in the corridor). In the valid stimuli, the images depicted a scene in which the three depth cues were globally consistent. In the invalid stimuli, the three depth cues did not globally match each other. The images were presented at the center of the visual field on a gray background with a visual angle of 15° × 15°.

### Experimental procedure

The experiments were conducted in two phases. In the first phase, the 21 subjected were trialed three times with the three context conditions in 5 sessions. In the first three session, only one of the three conditions was presented, while in the last two sessions, all conditions were presented. In the trials, an event-related design was used. In each run, trials for the valid and invalid stimuli were intermixed with an inter-stimulus-interval (ISI) in a predetermined order for each functional run as described previously (Vallesi et al., 2012). In each trial, stimuli were presented for 2 s after a 1 s fixation. The ISI between two consecutive context-valid or invalid stimuli was 0~3 s, between alternative context-valid or invalid stimuli was 9~12 s (Figure 2B). The subjects were instructed to continuously fixate on the central point of the objects to be judged and press the right or left button as quickly as possible when the context was valid or invalid. To eliminate the judgment errors due to task difficulty, the conditions were carefully matched for difficulty (for detail, see Results). The results obtained in the first phase were used to define the region of interest (ROI) for dynamic causal modeling (DCM) analysis. Each run contained 25 valid and invalid trials in the first phase.

In the second phase, the 21 subjects were stimulated twice in the same three sessions as in the first phase in an event-related design similar to that used in the first phase. Each run consisted of 150 trials, and during each trial, one stimulus randomly selected from the one of three stimulus sets was presented. The ISIs and the order of stimuli were predetermined based on the procedure used in the fMRI runs during the first phase. The time series data generated in the second phase were used for DCM analysis.

### Evaluation of the adequacy of the stimulus judgment

To evaluate the adequacy of judgment for visual contexts, we employed the procedure of the semantic differential (SD) method based on the data from a total of 300 stimuli as a psychometrically controlled scale of contextual validity (Paller et al., 2007).

**Figure 3:**
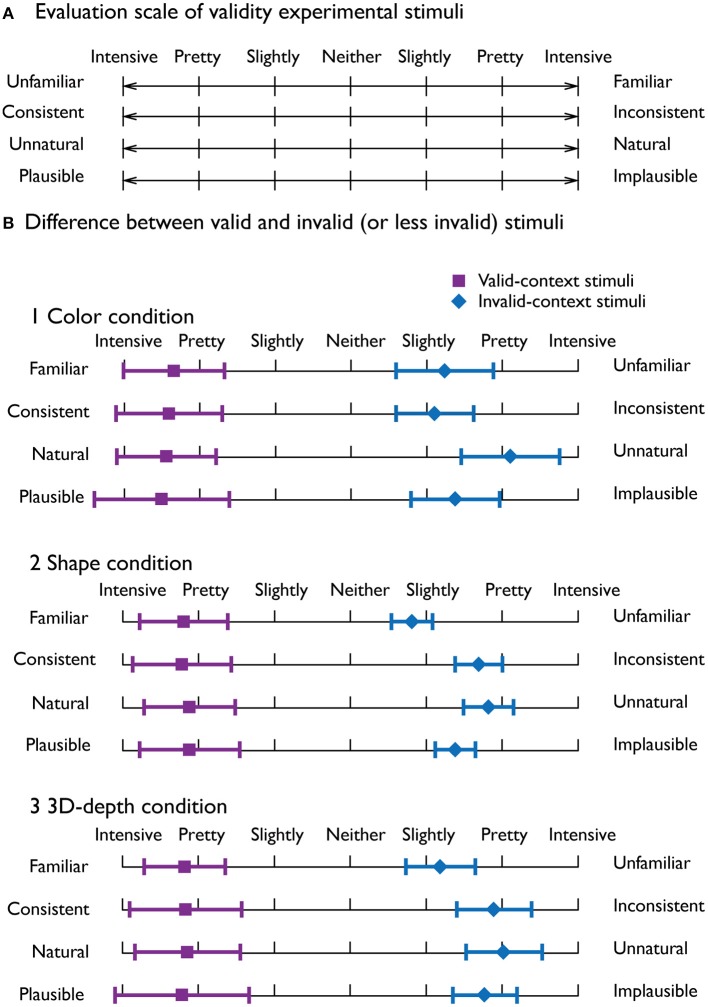
**Evaluation of the adequacy of the employed stimuli. (A)** Four seven-point bipolar adjective scale used to evaluate the adequacy of the visual stimuli. **(B)** Mean profiles for the valid-context stimuli (violet) and invalid-context stimuli (blue) for the three visual contexts. The error bars denote the standard deviation.

To rate the contextual validity, bipolar adjective scales with seven-point ratings were used (Figure 3A). Ten subjects were given the instructions that are essentially the same as those used by Osgood and Suci (1955). As shown in Figure 3B, it was clear that the judgment profiles were quite distinct between the valid and invalid stimuli, indicating that the characteristics of the stimuli were clearly recognized and the stimuli were appropriate.

### Experimental setup

Visual stimuli were generated on a personal computer (DELL desktop computer) and presented to the subjects via a custom-built magnet-compatible video system. The stimuli were projected onto a vertical screen positioned between an MR scanner and the projector. The subjects viewed the visual stimuli via a mirror above their eyes. The distance between the screen and the mirror was 190 cm. A color LCD projector (Epson, 1,024 × 768 pixels, 60 Hz refresh rate) was used to present the stimuli.

### fMRI experiment

Whole-brain fMRI scans were performed and acquired using a Philips 3.0T Intera scanner with gradient echo-planar imaging sequences (TR: 3,000 ms, TE: 50 ms, flip angle (FA): 90°, matrix size: 128 × 128) while the subjects were judging the context validity. The acquired slices were axial and parallel to the anterior-posterior commissure line (voxel size 1.8 × 1.8 × 4 mm). Thirty two slices were obtained from the bottom to top.

Standard whole brain structural scans (3D MPRAGE, sagittal acquisition, slice thickness 1.0 mm, in plane resolution 1.0 × 1.0 mm^2^; TR: 8.3 ms; TE: 4.6 ms; FA: 8°; SENSE factor: 2) were also obtained for the participants.

### Statistical parametric mapping

The fMRI data were analyzed using SPM8 (Wellcome Department of Cognitive Neurology, London, UK) implemented in MATLAB (MathWorks, USA). For each subject, the first four images were discarded and the remaining 143 images from individual runs were realigned to correct head motion and hemodynamic artifacts, using the mean image as a reference. The motion parameters generated in the spatial realignment indicated that the 21subjects moved less than 4mm on average. The realigned images were spatially normalized to the MNI (Montreal Neurological Institute) brain template (Ashburner and Friston, 1999). The normalized images were smoothed spatially with an isotropic 8 mm FWHM (full-width at half maximum) Gaussian kernel and re-sampled, resulting in voxels of 2 × 2 × 2 mm in size.

To identify the neural substrates that process the three visual contexts and to assess the significance of functional activation, we used a general linear model (GLM) analysis. In the first level (within-subject) analysis, the data were modeled voxel-wisely in GLM. The data were high-pass filtered (cut-off, 128 s) to remove low-frequency signal drifts and scaled down the number of images to 143 within each session. Non-sphericity of the error covariance was accommodated by an AR (1) (first-order autoregressive) model in which the temporal autocorrelation is estimated by pooling over supra-threshold voxels (Friston et al., 2002). Contrast images were created for each subject. There were 18 contrasts for the second-level group statistics: (1–6) valid vs. invalid and invalid vs. valid context of color (C), shape (S), and 3D-depth (D), which were used to define the difference between validity within conditions; (7–9) common activations of C, S and D, or C and S, or S and D, (10–12) common activations of valid and invalid context of C, S or D, (13) C vs. (S+D), (14) S vs. (C+D), (15) D vs. (C+S), (16) (S+D) vs. C, (17) (C+D) vs. S, (18) (C+S) vs. D. The contrast (con) images from the first-level analyses of all 21 subjects were used for the second-level analyses. To identify the areas activated both by valid and invalid stimuli in the C, S, and D conditions, or under all conditions, one-sample *t*-test analysis was carried out for each of the 9 con images. The resulting SPM (T) maps were then thresholded at *p* < 0.05 (cluster-level corrected, FWE; cluster forming threshold: *p* < 0.001, uncorrected). Among the 18 contrasts mentioned above, only statistics 7–13, 16–18 showed significant activations at the corrected threshold level. These results were then used to define the region of interest (ROI) for DCM analysis.

### Dynamic causal modeling

ROIs were defined by manually tracking the intersection of anatomical boundaries and significant functional activation informed by the results of the SPM analysis of the data. Dynamic causal models (Stephan et al., 2007) were fitted to subject-specific BOLD time series that were extracted from the data from the mixed sessions of fMRI runs. Because the exact locations of the activated areas vary across subjects, it is important to ensure that the models are comparable across subjects. To ensure this, we defined ROIs such that the extracted time series met a combination of anatomical and functional criteria. Anatomical boundaries were defined on the MNI template using automated anatomic labeling (AAL) toolbox (http://www.fmri.wfubmc.edu/cms/software#PickAtlas). ROIs for the respective areas were selected according to the results of the group analysis. We defined location of the individual (single-subject) ROIs by the local peals closest to the group coordinates in the respective cortical regions (Stephan et al., 2007).

For each subject and area, individual local peaks (*p* < 0.05 uncorrected) for the respective contrast were identified as described above for the mixed tasks (Eickhoff et al., 2009). Time series for these regions were then extracted as the first principal component for 15 most significant voxels in the target contrast centered around the individual peaks in a radius of 4 mm and adjusted for the effects of interest in SPM8.

### Construction of DCMs

**Figure 4:**
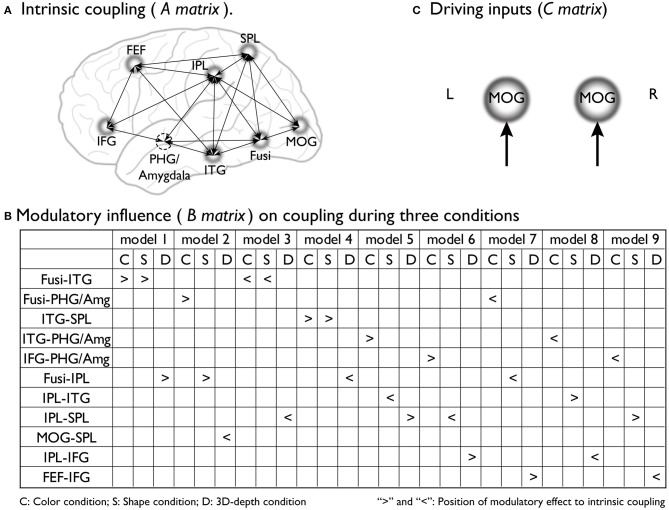
**Supposed intrinsic connectivity, modulatory influence and driving input in DCM models. (A)** Intrinsic coupling of proposed basic dynamic network models for the neural information processing during the judgments for C, S, and D conditions. **(B)** Modulatory influence of color, shape and 3D-depth on coupling during three conditions; **(C)** Photic inputs to the dynamic network.

To preform DCM, we assumed that the stimulus presentation directly evoked brain activity in early visual areas [middle occipital gyrus (MOG)18/19] regardless of the type of context. By using empirical evidence regarding the dorsal attentional network (Coull and Nobre, 1998; Nobre et al., 1998), the network for the associative context (Rowe et al., 2005), the neural pathway for the visual processing of depth (Kourtzi et al., 2003), and the neural substrates that process the three visual contexts identified through SPM analysis, we determined the regions, connections, and driving inputs to be used to construct the basic models of processing streams for the color, shape and 3D-depth contexts. Figure 4A shows models of the intrinsic connectivity defined for each condition; Figures 4B, **10** show the putative positions of modulatory influence to intrinsic connectivity for each model tested, and Figure 4C shows MOG, the entering point for photic input to the brain. For each condition, 9 models were tested for left and right hemispheres.

### Bayesian model comparison

To determine the optimal model structure based on the data observed from all subjects for each condition, the RFX Bayesian model selection (BMS) was used. The posterior probability model was obtained by taking the product of the model evidence from each subject-specific DCM and its prior model (Penny et al., 2004). The model evidence, as approximated by the free energy, depends not only on model fit but also on model complexity. Because the fixed effects group analysis can be distorted by outlier subjects, BMS was also implemented using a hierarchical Bayesian model to estimate the parameters of a Dirichlet distribution over the probabilities of all models considered (implemented in SPM8). These probabilities define a multinomial distribution over model space, enabling the computation of the posterior probability of each model given the data of all subjects and the models considered. For BMS, random effect tests were applied for each model using exceedance probability, which is used as posterior probability. Bayesian model averaging (BMA) was also carried out for each condition and hemisphere, because of no optimal model survived a posterior exceedance probability (EP) of 0.7. For example, an EP of 0.7 means that we can be 70% confident that a specific model has a greater posterior probability than any other models. In the case of only two competing hypotheses, the EP is particularly intuitive as it describes the confidence that a model is more likely than the other one. For the averaged model, the subject-specific intrinsic, modulatory and extrinsic effects were also tested using one-sample *t*-tests. In addition, photic inputs, modulatory influence and connection strengths were analyzed as described (Daunizeau et al., 2011).

## Results

### Behavioral results

**Figure 5:**
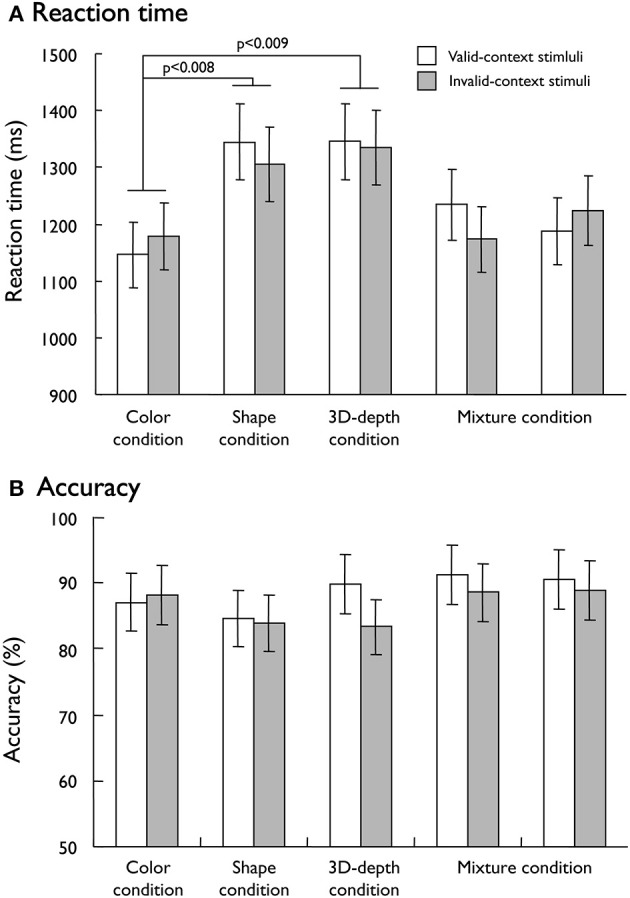
**Behavioral results responding to visual stimuli. (A)** Mean reaction time for the valid-context and invalid-context stimuli. **(B)** Mean accuracy rates for the valid-context and invalid-context stimuli.

To dissociate the neural networks responsible for visual processing of the three contexts (color, shape, and 3D-depth), we designed the conditions that were equalized for the difficulty of judging the contextual validity. To evaluate the validity of our task design, mean reaction times (RTs) and accuracy were used as measures of behavioral performance. As shown in Figure 5A, mean RTs in 5 fMRI sessions for valid- and invalid- stimuli were different among the three conditions (*p* < 0.001). However, no difference was observed between valid- and invalid- stimuli (*p* = 0.054). The subjects required significantly longer time to make a decision regarding contextual validity in the S (RT: 1,325 ± 67 ms, *t* = 10.406; *p* < 0.008) and D (RT: 1,340 ± 66 ms, *t* = 12.72; *p* < 0.009) conditions than in the C condition (1,162 ± 5 ms). The RT results indicated that it was not likely that the effect of the type of visual context resulted from stimulus complexity because D stimuli were more complex than S stimuli, but the RTs did not increase in proportion to the complexity. These results suggest that visual processing for S and D conditions is different from that for C condition. As for judgment accuracy, no difference was found among these conditions (Figure 5B). Analysis showed that neither the main effect of stimulus condition nor the interaction between contextual validity [*F*_(1, 205)_ = 1.15, *p* = 0.331] and stimulus condition was significant [*F*_(1, 205)_ = 0.097, *p* = 0.756], indicating that any differential effect in brain activity is unlikely to be due to task difficulty.

### Imaging results

#### Within-task activation

**Figure 6:**
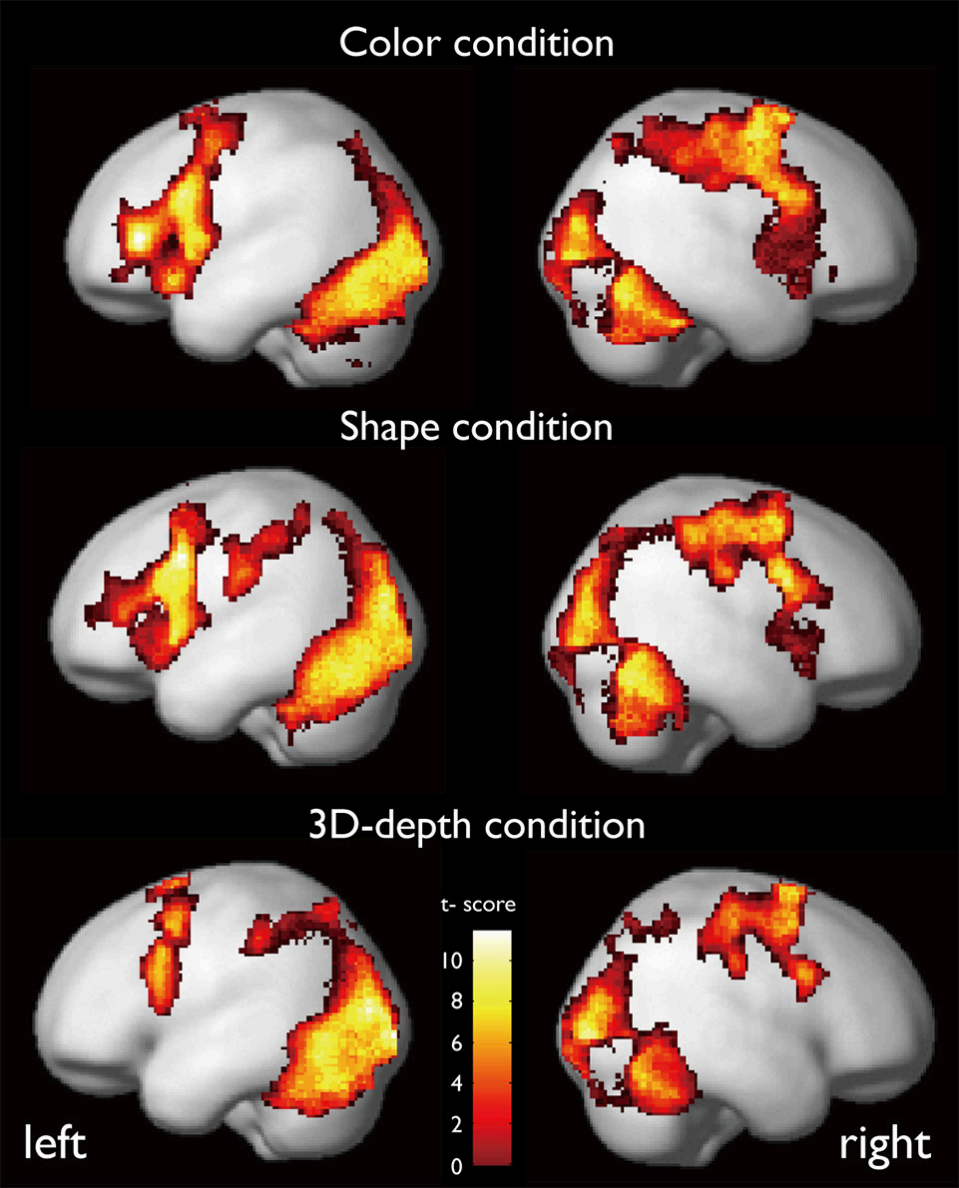
**Activation maps while judging the contextual validity for the three visual contexts**.

The regions that were activated during the judgment of contextual validity for each of the three visual contexts are shown in Figure 6 and Table 1 based on images obtained for valid and invalid stimuli. To examine the effect of the validity of visual context, we analyzed the data using the subtraction method based on a random-effects model. In six T-contrast (valid- vs. invalid-C, S, D, invalid- vs. valid-C, S, D) tests, we found no significant differences between the valid- and invalid stimuli at a threshold of *p* < 0.001 (uncorrected) at voxel level. Namely, for the visual contexts tested, the valid- and invalid-contexts activated the same neural regions.

**Table 1:** **Activated regions during judgments of contextual validity for visual contexts**.

**Cluster size (voxels)**	**Anatomical regions and BA**	***t*-score**	***x***	***y***	***z***
**COLOR CONDITION**					
5238	L inferior frontal gyrus BA 46	11.33	−44	34	8
	L inferior frontal gyrus BA 47	10.14	−32	24	−10
	L middle frontal gyrus BA 9	9.19	−46	14	32
	L inferior frontal gyrus BA 44	8.12	−48	10	16
	L putamen	7.02	−18	10	6
	L middle frontal gyrus BA 10	6.75	−36	40	20
	L middle frontal gyrus BA 6	5.69	−54	4	42
302080	R inferior frontal gyrus BA 47	10.49	32	22	−8
	L culmen	10.21	−28	−38	−26
	R fusiform gyrus BA 19	10.09	46	−72	−14
	R thalamus	10.02	12	−14	0
	L middle occipital gyrus BA 18	9.81	−42	−88	2
	L lingual gyrus BA 17	9.57	−12	−94	−12
	R middle occipital gyrus BA 18	9.47	30	−90	−2
	L superior frontal gyrus BA 6	9.46	−6	12	56
	L fusiform gyrus BA 19	9.11	−48	−66	−12
	R medial frontal gyrus BA 6	9.04	10	4	60
	R middle occipital gyrus BA 19	8.81	34	−90	14
	L parahippocampal gyrus amygdala	7.09	−18	−2	−18
	L precentral gyrus BA 6	6.88	−36	−6	64
	L middle frontal gyrus BA 6	6.75	−42	6	56
	L nodule	6.71	−2	−58	−30
	L thalamus	6.53	−10	−14	4
	L inferior semi-lunar lobule	6.07	−28	−62	−44
	L anterior cingulate BA 32	6.04	−8	30	24
	L lingual gyrus	5.85	−20	−66	0
**SHAPE CONDITION**					
316870	R middle occipital gyrus BA 37	9.56	58	−64	−8
	R fusiform gyrus BA 19	9.11	46	−70	−12
	R inferior occipital gyrus BA 18	8	42	−86	0
	R precuneus BA 7	6.84	16	−74	44
	L middle occipital gyrus BA 19	9.98	−26	−86	18
	L fusiform gyrus BA 37	9.47	−50	−56	−18
	L inferior occipital gyrus BA 17	8.3	−14	−92	−10
	L fusiform gyrus BA 18	8.18	−20	−90	−10
	L cerebellar tonsil	7.75	−34	−42	−34
	L superior parietal lobule BA 7	6.87	−26	−64	46
	L fusiform gyrus BA 37	6.83	−36	−48	−16
	L inferior parietal lobule BA 40	6.25	−42	−36	40
	L middle frontal gyrus BA 9	9.97	−44	8	36
	R inferior parietal lobule BA 40	9.15	42	−32	46
	R inferior frontal gyrus BA 9	8.69	48	8	28
	R middle frontal gyrus BA 6	8.65	42	4	58
	R inferior frontal gyrus BA 47	8.35	32	20	−6
	R precentral gyrus BA 4	8.31	32	−12	50
	R insula BA 13	8.21	36	14	6
	L insula BA 13	8.03	−34	20	0
	L inferior frontal gyrus BA 47	8	−34	26	−4
	L inferior frontal gyrus BA 46	7.86	−48	38	12
	L inferior frontal gyrus BA 44	7.81	−52	10	18
	R cingulate gyrus BA 32	7.73	12	16	44
	R postcentral gyrus BA 2	7.7	48	−22	34
	R postcentral gyrus BA 3	7.69	46	−16	58
390	R thalamus	8.05	12	−14	8
	L thalamus	5.84	−14	−10	8
**3D-DEPTH CONDITION**					
292240	L middle occipital gyrus BA 18	11.48	−28	−94	10
	L middle occipital gyrus BA 19	11.26	−32	−94	8
	R middle occipital gyrus BA 18	10.98	28	−90	2
	L fusiform gyrus BA 37	10.14	−52	−64	−12
	R medial frontal gyrus BA 6	9.73	10	4	60
	R fusiform gyrus BA 37	9.49	52	−70	−12
	L fusiform gyrus BA 37	9.1	−42	−64	−12
	R middle frontal gyrus BA 6	9.07	34	2	48
	L lingual gyrus BA 17	9.05	−16	−94	−14
	L superior frontal gyrus BA 6	9.05	−2	14	50
	L inferior parietal lobule BA 40	8.94	−38	−44	42
	L medial frontal gyrus BA 8	8.84	−4	18	46
	L precentral gyrus BA 9	8.82	−36	8	36
	L middle frontal gyrus BA 6	8.55	−42	4	48
	L inferior frontal gyrus BA 44	7.86	−48	10	22
	L inferior frontal gyrus BA 46	7.12	−50	38	10
	L precuneus BA 7	6.7	−8	−74	48
	L culmen	6.64	−8	−32	−8
	R brainstem	6.26	8	−26	−6

### Common activation pattern

To determine regions that were commonly activated during the judgments of validity across the different visual contexts, we used conjunction analysis in SPM8 to identify common activation regions (CARs) as follows:

#### CAR for C, S, and D conditions

**Figure 7:**
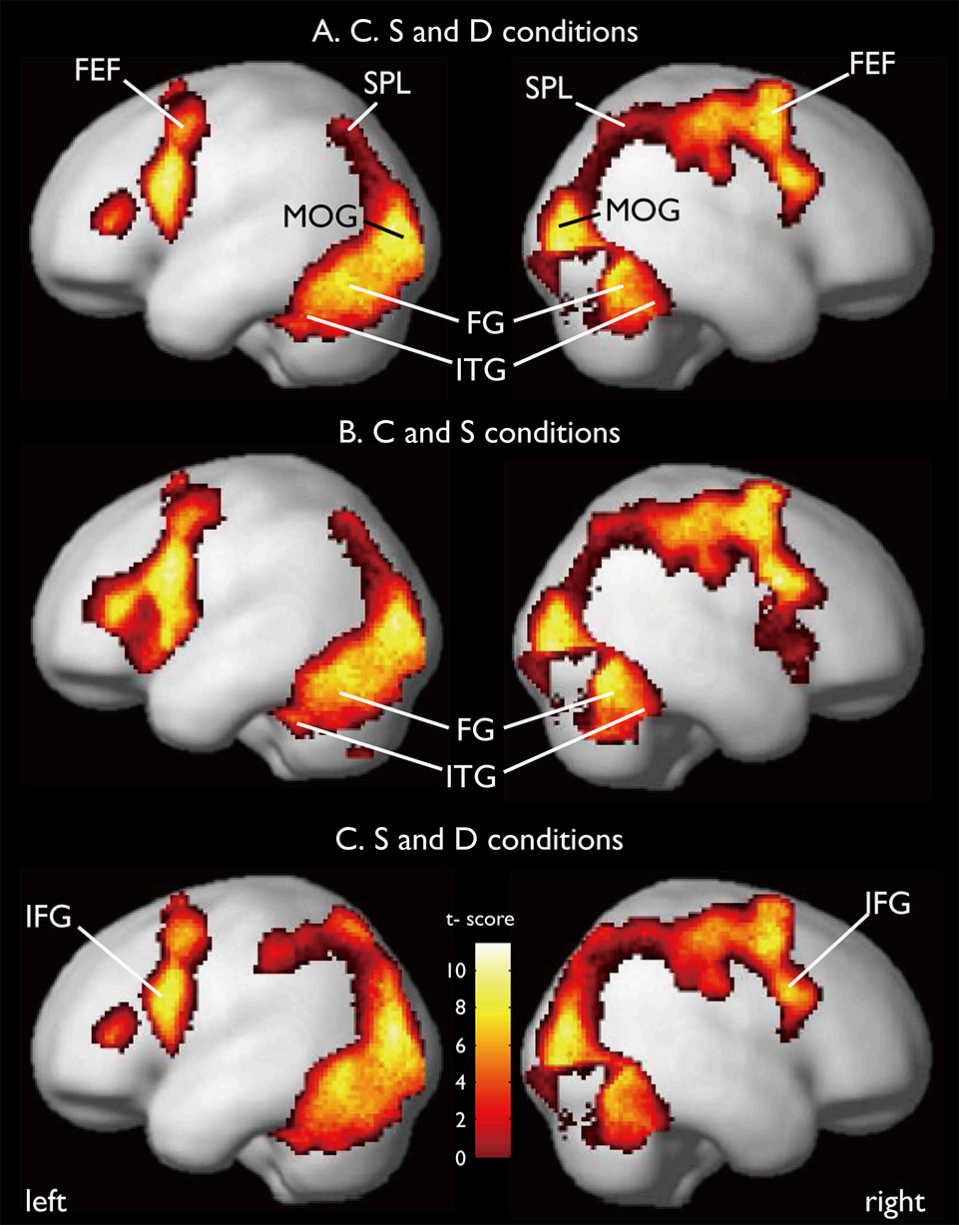
**Common regions of activation while judging more than one conditions. (A)** Common activation areas for C, S, and D conditions. **(B)** Common activation areas for C and S conditions. **(C)** Common activation areas for S and D conditions. C, color condition; S, shape condition, D, 3D-depth condition.

CARs during the judgments of the three contexts are shown in Figure 7. The color context and feature (shape and 3D-depth) contexts elicited the activations in four common regions. Based on the MNI template, the areas and coordinates of the activated areas were right MOG (*x* = 30, *y* = −88, *z* = 4), right SPL (*x* = 28, *y* = −62, *z* = 44), right FEF (*x* = 42, *y* = 0, *z* = 54), left MOG (*x* = −36, *y* = −88, *z* = 4), left SPL (*x* = −28, *y* = −64, *z* = 42), left FEF (*x* = −44, *y* = 8, *z* = 30), right FG (*x* = 50, *y* = −70, *z* = −12), left FG (*x* = −52, *y* = −70, *z* = −18).

#### CAR for C and S conditions

CARs identified for C and S conditions are shown in Figure 7B which include the inferior temporal gyrus (ITG) and FG. The MNI coordinates of the clusters were *x* = 56, *y* = −64, *z* = −4 for right ITG (BA37), and *x* = −54, *y* = −60, *z* = −10 for left ITG (BA37).

#### CAR for S and D conditions

As shown in Figure 7C, CARs identified for S and D conditions were IFGs located in the right hemisphere [IFG (BA45), *x* = 56, *y* = 12, *z* = 24] and left hemisphere [IFG (BA45): *x* = −60, *y* = 16, *z* = 22].

#### Specific activation regions (SAR) for color

**Figure 8:**
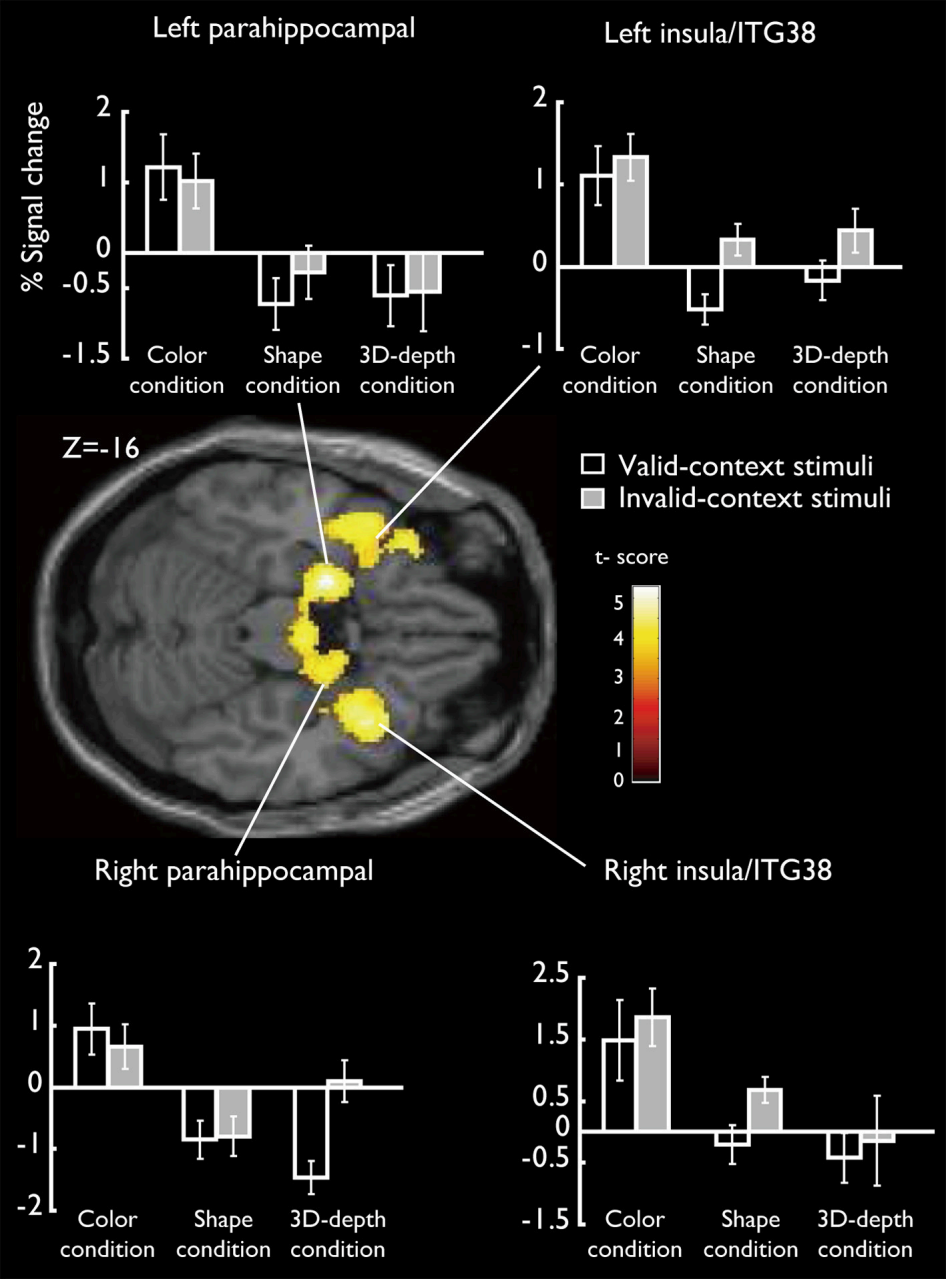
**Specifically activated cortical regions during the judgment for C condition and their signal changes for each condition**. Parahippocampal and ITG (BA38) were significantly activated (cluster-level corrected, FWE; cluster forming threshold: *p* < 0.001, uncorrected).

SARs for color were shown in Figure 8. These regions were not activated by the other two object features. SARs identified included the parahippocampal gyrus/Amygdala(PHG/Amg) and frontal polar portion of the superior temporal gyrus (STG/BA38) symmetrically located in both hemispheres. The MNI coordinates of these regions were right PHG (*x* = 18, *y* = 0, *z* = −22), right STG (BA38) (*x* = 38, *y* = 16, *z* = −20), left Amg (*x* = −18, *y* = −2, *z* = −18), left STG (BA38) (*x* = −44, *y* = 14, *z* = −20). The 4 ROIs were further defined as spheres with a radius of 8 mm to extract the signal changes for each condition.

#### SAR for shape and 3D-depth

**Figure 9:**
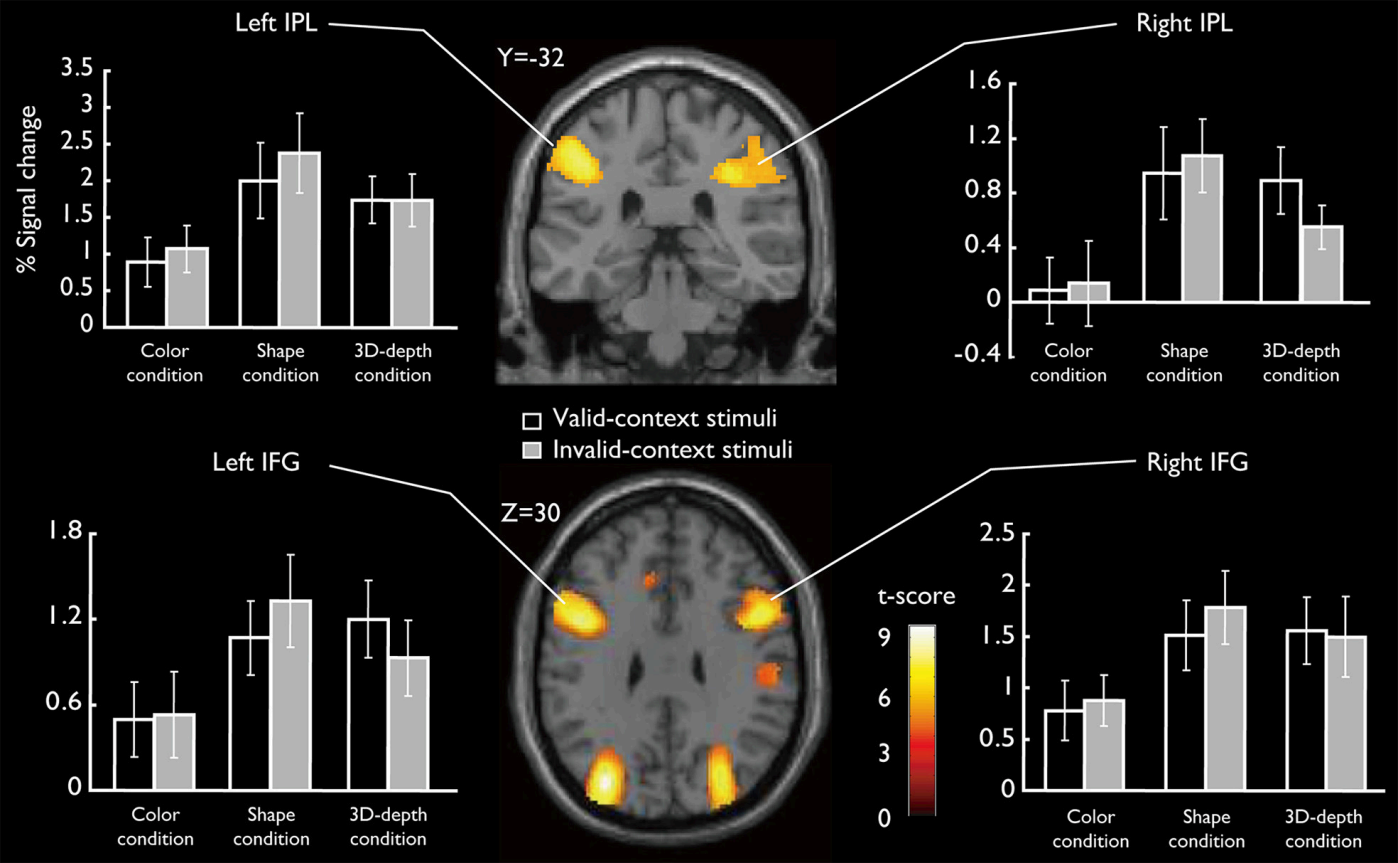
**Specifically activated cortical regions during the judgment for S and D conditions and their signal changes for each condition**. Bilateral IPL and IFG were significantly activated (cluster-level corrected, FWE; cluster forming threshold: *p* < 0.001, uncorrected).

SARs for shape and 3D-depth are shown in Figure 9, and they were right IPL (BA40) (*x* = 42, *y* = −32, *z* = 46), left IPL (BA40) (*x* = −42, *y* = −38, *z* = 42), right IFG (*x* = 38, *y* = 0, *z* = 48) and left IFG (*x* = −40, *y* = 4, *z* = 48). The 4 ROIs were further defined as spheres with a radius of 8 mm to extract the signal changes for each condition.

#### DCM results

**Figure 10:**
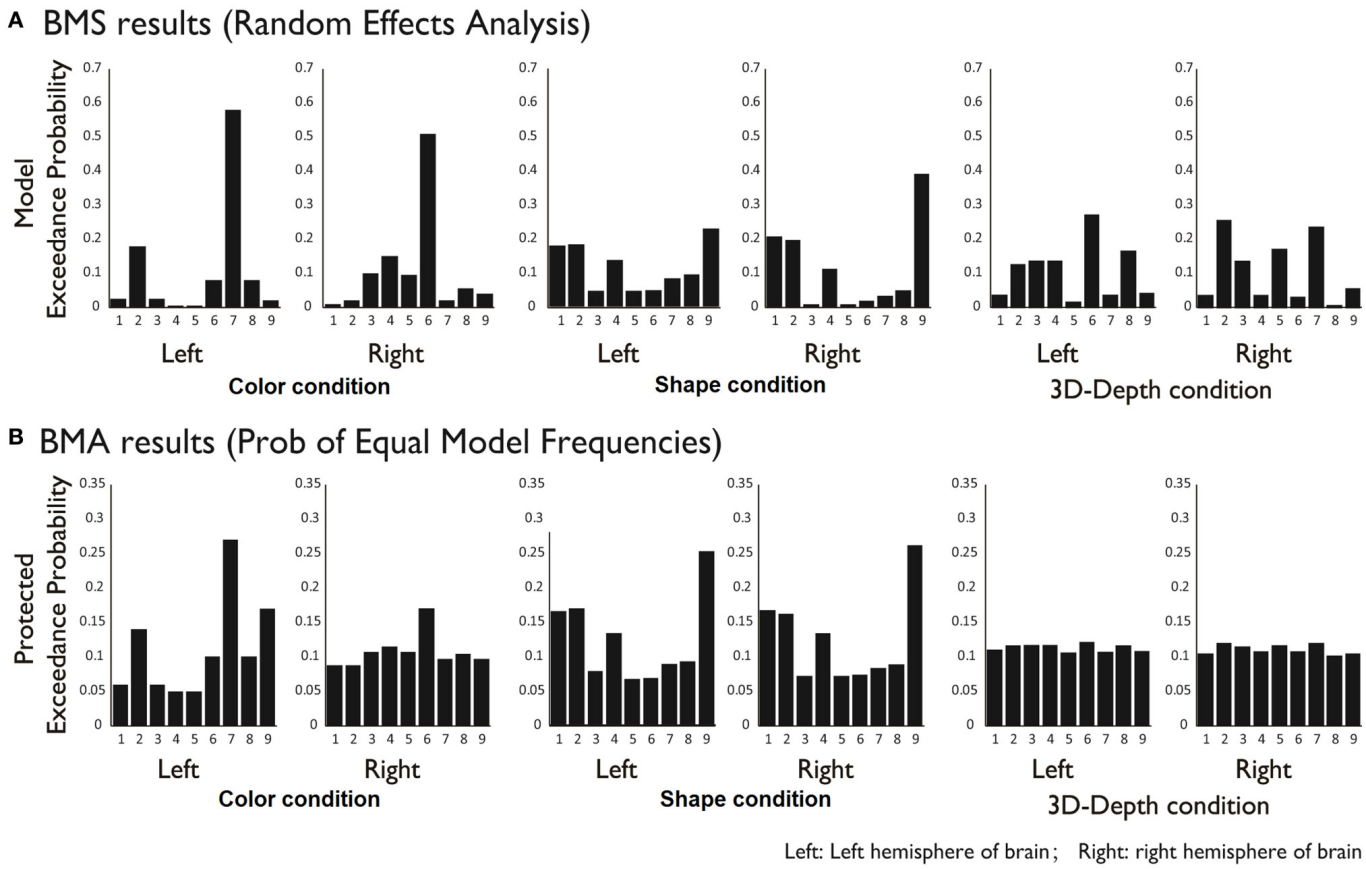
**BMS (A)** and BMA **(B)** results for C, S, and D conditions for 9 modes.

To reveal the dynamics in network for each condition, we carried out the DCM analysis and the results are shown in Figure 10A (BMS-RFX) and Figure 10B (BMA) for each hemisphere. BMS-RFX results showed that, for each condition and brain hemisphere, although exceedance probability for each model was different, it did not exceed 0.7, indicating that the optimal model could not be selected based on individual candidate model. Hence, the averaged effect of 9 models for each condition and brain hemisphere was calculated using BMA analysis. The BMA results showed that, for color and shape conditions, the contribution of candidate models to the DCM models was different. Models 7 and 9 had greater contribution for color condition and model 9 had greater contribution for both shape and hemisphere. However, for 3D-depth condition, all models showed similar effect in the DCM models.

**Figure 11:**
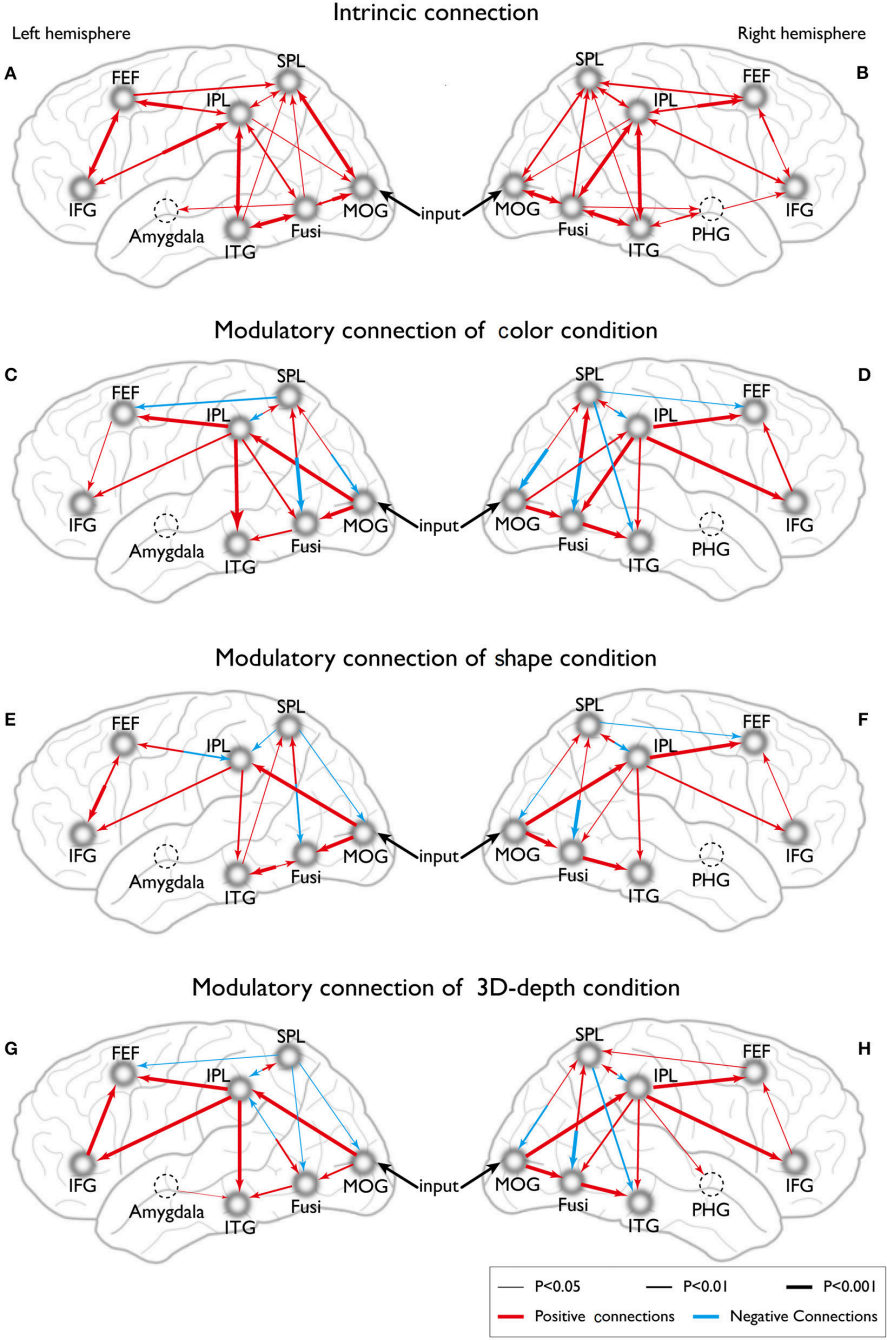
**Dynamic connections for the intrinsic and neural processing of the three visual contexts. (A,B)** Intrinsic connections of left and right hemispheres. **(C,D)** Connections of left and right hemispheres in C condition. **(E,F)** Connections of left and right hemispheres in S condition. **(G,H)** Connections of left and right hemispheres in D condition.

Eight models of activated sites were constructed for the processing of color (Figures 11C,D), shape (Figures 11E,F) and 3D-depth (Figures 11G,H). As shown, the visual stimulus inputs for color entered the system by directly activating MOG18/19. The induced activation then spread along the reciprocal connections between MOG and FG and between MOG and SPL. The activity was further expended along the reciprocal connections among SPL, FG, FEF, and ITG. The reciprocal connections between FG and SPL, SPL and FEF, and SPL and FEF were based on previous studies of the dorsal attentional network (Coull and Nobre, 1998; Nobre et al., 1998). The induced activation in FG was found spread to ITG following the method used for revealing the function of PHG (Epstein and Ward, 2010).

To define the connections for shape processing, similar procedure was adopted with slight modifications in the basic connectivity layout. Here, we focused on connections among FG, IPL, IFG, and ITG because these areas had previously been implicated to contribute to visuospatial (object and depth) tasks (Chun and Jiang, 1998; Martínez et al., 1999; Claeys et al., 2004; Hayes et al., 2004; Olzak and Laurinen, 2005; Bar et al., 2006). As in case of color, the visual stimulus inputs entered the system by directly affecting MOG18/19. The induced activation then spread along the reciprocal connections between MOG and FG, and between MOG and SPL, and expanded to SPL, FG, IPL, IFG, FEF, and ITG along their reciprocal connections (Figures 11E,F).

Similarly, the connections were identified for 3D-depth (Figures 11G,H), where the visual stimulus inputs entered the system by directly activating MOG (BA18/19) and were then transmitted along the reciprocal connections between MOG and FG as well as along the reciprocal connections between MOG and SPL, and spread to SPL, FG, IPL, IFG, and MFG.

## Discussion

Our findings have demonstrated that the mode of functional connectivity in the cortical pathways as well as the pattern of activation in these pathways are distinct during the processing of different visual contexts for the first time. We have identified CARs for color, shape and 3D-depth, such as FG, SPL, and IFG, and visual context—specific activation clusters in the temporal and prefrontal cortices. Our analysis shows that processing of the association context can be distinguished by the pronounced activation of ITG (BA37), STG (BA38), and PHG (Figures 6A, 8); in the processing of shape and 3D-depth contexts, IPL, ITG (BA37) and the IFG (BA45) in the middle prefrontal gyrus are activated. These results extend previous neuroimaging evidence that the post-rolandic parietal and temporal visual cortices are important for encoding shape and 3D-depth context, whereas the temporal and parahippocampal cortices are critical for establishing the association context (Epstein and Ward, 2010). These results are also consistent with the view that contextually modulated-attentional control signals affect the neural activation in visual cortical areas (Womelsdorf et al., 2006; Roberts et al., 2007).

These new findings reveal the dynamic properties of a global functional network architecture that is characterized by two coexisting organization principles (i.e., functional segregation and integration). This architecture may play a role in the active maintenance of visual information against degradation from the mutual information that is represented within the cortices that modulate different types of information processing, where the posterior cortex is related to feature processing, the inferior cortex controls the processing of objects, the parietal cortex is involved in the processing of 3D structure, and the parahippocampal cortex affects association processing (Figure 11). As such, the inferior PFC may play a key role in controlling differential activations between the visual pathways that are involved in the visual processing of different types of context.

### Function of PFC in the contextual processing of visual stimuli

Traditionally, PFC function in humans has been studied using a processing approach (Duncan, 2001), which assumes that cognition by the PFC can be described in terms of performance without specifying the representation that underlies these processes. In brain imaging and electrophysiology, comparisons between conditions have been made at various levels and have shown focal peaks of activity at low demand, which then evolve into a pattern of largely overlapping activity at higher demand (Scalaidhe et al., 1999; Haxby et al., 2000, 2001; Duncan, 2001; Wallis et al., 2001). On the other hand, based on a cascade model of cognitive control, Koechlin et al. (2003) showed that the engagement of prefrontal regions along the poster anterior axis is not primarily based on condition demands, such as relational complexity or memory load, instead, it is based on the temporal structure of the representations underlying executive control. This favors the representational approach, which seeks to establish the motion that information is stored in the PFC (Goldman-Rakic, 1995a,b). Contextual processing critically relies on the active maintenance of abstract (rule-like) representations in the PFC that guides the processing in the posterior cortex (Rougier et al., 2005). Thus, our results demonstrate that distinct regions in the PFC represent visual information in different manners depending on whether or not the regions are carrying out active maintenance or rapidly updating representations of information (Figures 11A,B).

### Effective connectivity between the visual and PFC

A functional brain imaging study revealed that there are large-scale spatial organizations for specialization within the visual pathways (Haxby et al., 2000), and network analyses based on the anatomical links between brain regions have demonstrated the functional connectivity along the cortical visual pathways (McIntosh et al., 1994). In contrast to such large-scale analyses at successive hierarchical levels in the visual pathways, analysis of the latency of visual responses in cortical areas has yielded a somewhat different picture than what is expected on purely anatomical grounds. Such analyses have consistently found that the neurons in MT and FEF areas are activated almost as rapidly as the neurons in area V1 (Schmolesky et al., 1998; Lamme et al., 2000; Capalbo et al., 2008). Our study showed that the activated regions are converged at FEF and are specific for different types of visual information within the PFC. These results favor the evidence based on visual response latency that visual information converges at the posterior part of the PFC. Our results (Figures 11A,B) indicated that there is at least one neural site for the convergence of different types of visual information in the posterior part of the PFC.

### Activation of PHG/Amygdala

DCM analysis showed that in the optimal DCM for the association context, color context had a modulatory effect on the path from FG to PHG/Amygdala. Since PHG is important for association context (Kyle et al., 2015; Boccia et al., 2016), Bar and colleagues proposed that the PHG (including the parahippocampal place area (PPA)) encodes visual context information, which they defined as information about which objects “typically co-occur in the environment around us” (Bar et al., 2008). Along this line, parahippocampal responses to visual scenes are proposed to reflect the activation of a “context frame” representation that includes information about which objects typically appear in that context and where they are likely to be located relative to each other. These authors further proposed that there is a division of labor within the PHG such that the anterior PHG primarily encodes information about the identities of the typical objects, whereas the posterior PHG (i.e., the PPA) primarily encodes information about their typical locations. In our study, we observed the activation of the bilateral anterior PHG/AMG during color conditions, suggesting that they play a role in encoding information about the identities of the typical objects, that is, a typical association between an object and its color in the natural environment.

According to the results of DCM analysis, we found that the connection strength between the FG and PHG was significant only in intrinsic connections. This may be due to 2 possible methodological issues in our experiments that might have affected PHG activation. The first was low rate of stimulus presentation at 0.33–0.5 Hz, which might lead PHG to reflect mental imagery of scenes rather than a rapid activation of contextual representations as postulated (Bar, 2004). The second was that the valid- and invalid-context stimuli might differ with respect to their low-level visual properties, which are known to affect PHG activity (Levy et al., 2001; Rajimehr et al., 2011).

### Limitations

In this study, we used three visual contexts to reveal the dynamics in effective neuronal connection in the brain networks and have found specific networks for each condition. However, because we modeled the basic visual function and higher level functions such as attention and memory in the DCM models, only intra-hemispheral models were presented and discussed (since the DCM analysis allows to use only 8 ROIs in one model). Further studies are need to elucidate how inter-hemisperal interactions occur in the processing of visual contexts.

## Conclusion

Our results show that brain activations related to color context processing occur specifically in temporal cortex, including PHG/Amg, while CRAs for the three types of context processing are MOG, FG, ITG (BA37) and the dorsal attention pathway. We propose that these contexts are processed in regions in the visual cortex and dorsal attention pathways. Based on the regional activations, 9 models for each type of context processing are developed for DCM analysis. The BMA results show that color context is processed from MOG to not only the dorsal but also the ventral visual pathway, while for shape and 3D-depth context processing, brain regions other than PHG are involved.

## Author contributions

QW, ST, HS, CL, and JW designed experiments. QW, ST, HS, QG, and JW conducted experiments. QW, YE, CL, and JW analyzed data. QW, CL, JW, and TY wrote manuscript. All authors approved the manuscript.

### Conflict of interest statement

The authors declare that the research was conducted in the absence of any commercial or financial relationships that could be construed as a potential conflict of interest.
